# The validity and reliability of the Arabic version of the short form of neurogenic bladder symptoms score in patients with spinal cord injury

**DOI:** 10.1186/s13018-023-03956-6

**Published:** 2023-06-27

**Authors:** Younes A. Khadour, Meng Zheng, Fater A. Khadour

**Affiliations:** 1 Department of Orthopaedic Surgery, Tongji Hospital, Tongji Medical College, Huazhong University of Science and Technology, Wuhan, 430030 China; 2 Department of Rehabilitation, Faculty of Medicine, Al Baath University, Homs, Syria; 3 Department of Physical Therapy, Health Science Faculty, Al-Baath University, Homs, Syria; 4 Department of Rehabilitation, Tongji Hospital, Tongji Medical College, Huazhong University of Science and Technology, 1095#, Jie-Fang Avenue, Qiaokou District, Wuhan, 430030 Hubei China; 5 Department of Physical Therapy, Physical Therapy Department for Neuromuscular and Neurosurgical Disorder and Its Surgery, Cairo University, Cairo, Egypt

**Keywords:** NBSS-SF questionnaire, Quality of life, Validity, Reliability, Neurogenic bladder, Arabic validation, Spinal cord injury

## Abstract

**Background:**

The Neurogenic Bladder Symptom Score-Short Form (NBSS-SF) evaluates the impact of disease-specific symptoms on the quality of life in individuals with neurogenic bladder (NB). There is no data on the validity and reliability of the NBSS-SF questionnaire in the Arabic language, so this study aimed to examine the psychometric characteristics of the Arabic NBSS-SF in patients with spinal cord injury (SCI).

**Methods:**

International standards were followed when culturally adapting the questionnaire. The Arabic version was conducted in patients with neurogenic bladder caused by SCI twice within a 14 day period. Psychometric properties such as content validity, construct validity, internal consistency, and test–retest reliability were tested. Internal consistency and test–retest reliability was evaluated using Cronbach’s alpha, and the Intraclass Correlation Coefficient (ICC), respectively. Construct validity was assessed by comparing the NBSS-SF with the Short Form (SF-12) and the Qualiveen questionnaire.

**Results:**

One hundred and one patients with SCI participated in the study. The internal consistency for the overall NBSS-SF score (Cronbach’s α of 0.82) and for each subdomain was variable (urinary incontinence 0.84; storage/voiding 0.72; consequences 0.57). ICC was 0.91 for the overall score and 0.94 for the urinary incontinence subdomain, 0.72 for storage/voiding, and 0.90 for consequences. The correlation analysis showed that the Arabic version of NBSS-SF has good construct validity.

**Conclusion:**

Our results showed that the Arabic version of NBSS-SF is a valid and reliable instrument for evaluating NB symptoms in the Arabic population suffering from SCI.

**Supplementary Information:**

The online version contains supplementary material available at 10.1186/s13018-023-03956-6.

## Introduction

Neurogenic Bladder (NB) is a set of symptoms and complications that characterizes a variety of pathological conditions, such as spinal cord injury (SCI) [1]. These complications comprise urinary tract infections, urinary incontinence, renal failure, and bladder stones, which could decrease the quality of life (QOL) and have a significant socio-economic impact [2, 3].

More than 80% of patients with MS and SCI experience bladder dysfunction or lower urinary tract symptoms [1, 4]. These types of patients require special care in managing their neurogenic bladder, which may include recurrent intermittent bladder catheterization or an indwelling catheter. Generally, all the current interventions and treatments attempt to enhance patients' QOL who suffer from the neurologic bladder [4, 5]. Therefore, using generic questionnaires can lead to highly inaccurate measurement of urinary problems, which impedes treatment [2]. Patient Reported Outcome Assessments (PROs) are useful methods for describing symptom burden and health-related quality of life (QoL), and they are gradually becoming more important in clinical decision-making [6]. The selected PRO should be applicable and appropriate for that particular population and the desired outcome, while it should also be validated for the language that the target population speaks [5]. Different types of questionnaires were developed for evaluating urinary disorders in varying clinical conditions [7, 8], such as Qualiveen [9] and Urinary Symptoms Profile (USP) [10]. They were designed to assess health-related QOL for neurologic bladder patients with SCI and later validated for patients with MS [11].

To meet the need for an instrument to evaluate urinary-specific QOL in terms of symptom burden in 2013, Welk et al. developed the Neurogenic Bladder Symptom Score (NBSS) [12]. The Neurogenic Bladder Symptom Score (NBSS) is a questionnaire used to evaluate the symptoms of a neurogenic bladder. It includes 24 questions; the first classifies patients using bladder management methods. The following 22 questions evaluate three domains: voiding, incontinence, and consequences, and the last question addresses the overall quality of life [12, 13]. Then, in 2020, Welk et al. developed a short version of NBSS (NBSS-SF) that includes 10 items evaluating the three same domains as the NBSS original version [14].

NBSS-SF has the benefit of evaluating three domains (urinary incontinence, bladder storage, and voiding) and assessing the quality of life that is significant for NB patients. This scale also has two questions about lower urinary tract symptoms (LUTS) and the treatment efficiency for LUTS. The NBSS has been validated in different languages, such as Portuguese [15] and Russian [16], Turkish [17], and Greek [18], in addition, it has been evaluated in people with MS [18], SCI [19], and adults who experience cerebral palsy [20] as well as the NBSS-SF, has been validated in French language [21].

The use of validated questionnaires is promoted in contemporary medicine to create reliable data for clinical research and to compare the outcomes of clinical research performed across different countries, populations, and cultures [22]. Considering the lack of appropriate PRO to assess patients with the neurologic bladder in Arabic, this study aimed to translate and validate the short form of Neurogenic Bladder Symptom Score NBSS-SF in Syria and evaluate its characteristics among Arabic-speaking SCI patients.

## Methods

### Forward and back translation and cross-cultural adaptation

The translation of the NBSS-SF questionnaire was carried out based on guidelines for self-reported measures provided by Beaton et al. [23]. Permission to adapt the instrument was granted by the NBSS-SF author. This study was approved by the Ethics Committee of Al Baath University Institutional Review Board Consent Letter. In accordance with the Helsinki Declaration, all the patients voluntarily agreed to participate in the study, and their written consent was obtained. For forward translation, was carried out by performed by two bilingual translators independently, whose native language is Arabic, to translate the original version of the questionnaire (English) to the target (Arabic) language. The bilingual translators then gathered to evaluate each translated word, term, and sentence in the questionnaire, and the majority's decision resulted in the development of an initial Arabic form. Afterward, the initial Arabic form was back-translated to the original language (English) by an English native speaker, fluent in Arabic with no medical background and blind to the study purpose. The next step, the authors were convened to review the English back-translation to reveal any inconsistencies with the Arabic version. The results were evaluated by a committee of bilingual members composed of two doctors who specialized in neurorehabilitation (AG, YR), one expert in the neurologic bladder (AM), and one urogynecologist (SF).The items of the instrument were evaluated to determine the clinical relevance and suitability for use in the particular patient population, in addition to determining the semantic, idiomatic, and conceptual equivalence of each version item [23]. The proposed modifications were carried out only when the percentage of agreement was about 90% [23]. All stages of the study were reported to the original study's author. The last step was a pilot study of the Arabic version of the NBSS-SF among 15 Syrian patients with SCI (7 women and 8 men) in order to evaluate their understanding of the items of the questionnaire and whether the alternatives were clear. Minor adjustments were performed by the committee according to the comments of the 15 participants. After adjustments, the final Arabic version was concluded.

### Setting and recruitment of the patients

A total of 101 participants from 4 neurorehabilitation centers in 2 Syrian provinces (Damascus and Lattakia) were recruited for this study between December 2021 and June 2022. All the participants eligible for inclusion were ≥ 18 years, were diagnosed with spinal cord injury, and could fluently read and speak the Arabic language. Exclusion criteria were: (i) patients with urologic surgery in the last 12 months, (ii) a recent change in the general health situation (iii) a change in the treatment symptoms of their NB or active urinary infection in the last 30 days. The criteria used in this study were the same ones that were adopted by the developer of the NBSS-SF questionnaire [14].

Before participation. In addition, any participants who represent a change in bladder and urinary tract function between the first and second visit will be excluded.

#### Instrument

All patients completed a packet containing the NBSS-SF, demographic and clinical information, Short Form-12 (SF-12) and Qualiveen QoL questionnaire.

The NBSS-SF questionnaire is a self-reported instrument used to evaluate the symptoms of the lower urinary tract and assess of consequences of NB. The 10-item scale assesses the quality of life (QOL) through three domains: incontinence (three items), storage and voiding (three items), and consequences (two items). Two further questions, the first one is related to the method of bladder management, and the second one is about how the current bladder management method affects the quality of life by using a scale ranging from 0 points (pleased) to 4 points (unhappy). The NBSS-SF ranges from 0 to 28, with a higher score indicating more severe symptoms [14].

All the participants were asked to re-answer the questionnaire within 10–15 days to evaluate test–retest reliability. The time between the two tests was short enough to prevent any changes in the patients' bladder management strategies or symptoms and long enough to reduce the chance of remembering the questions.

The Short Form-12 is an abbreviated version of the original 36-item QOL questionnaire, available in Arabic [24]. It comprises 12 items with a mental component summary (MCS) and a physical component summary (PCS). Possible scores for mental and physical health-related QOL subscales range from 0 to 100 points, and a higher score is interpreted as better QOL.

The Qualiveen is the instrument specific for urinary and bladder disorders in patients with neurological conditions, such as multiple sclerosis and spinal cord injury, available in Arabic [25]. It consists of 30 items; it contains four domains: (1) Limitations/Inconvenience, (2) Constraints/Restrictions, (3) Fears, (4) Feelings/Impact on Daily Life. This instrument's domains comprise a combination of emotional, physical, and social problems regarding bladder dysfunction. This questionnaire employs a 5-point Likert scale. It is possible to compute an overall (average) score in which the higher scores on this questionnaire indicate lower quality of life.

### Psychometric properties

In our study, the NBSS-SF questionnaire's psychometric characteristics were assessed under the guidelines of the process of cross-cultural adaptation [26]. Reliability is the degree to which a measurement is free from measurement error [27]. Internal consistency, test–retest reliability, and measurement characteristics were all assessed in this study. Internal consistency indicates how closely items from the same subscale or domain correlate. It is measured by Cronbach’s alpha a value greater than 0.70 is considered a good internal consistency [28].

The test–retest reliability aims to compare scores collected on two different occasions to demonstrate the data's reproducibility. The test–retest reliability of the NBSS-SF questionnaire was measured using the visit 1 and 2 NBSS-SF scores. The Intraclass Correlation Coefficient (ICC) was used to evaluate the outcomes. Since we applied ICC for the Test–retest reliability study, we selected the Two-way mixed effect model according to the guideline of selecting and reporting intraclass correlation coefficients for reliability research [29]. A correlation greater than 0.7 is considered strong [28].

Validity refers to the accuracy of a measure (whether the results really do represent what they are supposed to measure) [27]. In our study, we assessed the measurement characteristics of content and construct. Construct validity indicates the ability of an instrument to measure the fundamental concept it is looking to search for [28]. Construct validity in this study was evaluated by Spearman’s rank correlation comparing the Qualiveen questionnaire and the Short Form-12 with item number 2 from the NBSS-SF questionnaire. Content validity refers to the degree to which an assessment instrument is relevant to, and representative of, the targeted construct it is designed to measure and can be evaluated by experts in the field who may determine whether the suggested questions on the scale are pertinent and comprehensive to the phenomenon to be evaluated [28]. In this study the content validity was evaluated by two doctors (AM, SF), one expert in the neurologic bladder, and the other a urogynecologist who were members of the expert committee responsible for the translation of the NBSS-SF into Arabic. The sample size estimations were calculated according to the minimum requirement of at least 10 patients per question, as recommended in the literature [30]. The NBSS-SF questionnaire comprised 10 items, so we recruited a minimum of 100 patients.

### Statistical analysis

Baseline characteristics are reported as percentages when referring to categorical data and mean, and standard deviation was used for continuous data. The Kolmogorov–Smirnov test was used to determine the normal distributions of the variables. The internal consistency of the NBSS-SF was evaluated using Cronbach's alpha, with a value of 0.7 or greater considered acceptable (less than 0.50 = unacceptable; 0.6–0.5 = poor; 0.7–0.6 = questionable; 0.8–0.7 = acceptable; 0.9–0.8 = good; greater than 0.90 = excellent) [31] All the items of the NBSS-SF were included in the reliability test, except the first item, which assesses the bladder management methods. Test–retest reproducibility was calculated using the intraclass correlation coefficient (ICC). The construct validity of the NBSS-SF questionnaire was evaluated using appropriate analysis of the correlations with the Qualiveen questionnaire and Short Form-12; we used Spearman’s rank correlation because the data were not normally distributed. The results are presented with a 95% confidence interval (CI). The strength of the correlation was interpreted as follows: (0.00)–(± 0.10) = negligible; (± 0.10)–(± 0.39) = weak correlation; (± 0.40)–(± 0.69) = moderate correlation; (± 0.70)–(± 0.89) = strong correlation; and (± 0.90)–(± 1.00), very strong correlation [32]. All statistical analyses were analyzed using SPSS Version 23.0.and the level of significance was fixed at 0.05.

## Results

A total of 101 patients participated in the study, 73 patients (71.3%) were men, aged between 18 and 66 years, mean of 38.4 ± 11.2. Table 1 shows the participants' demographic information and clinical characteristics, in addition to the time of neurological disease, level of injury, educational level, and type of bladder management.

**Table 1:** Demographic and clinical characteristics

Demographic and clinical characteristics	n (total = 101)
*Gender*	
Male	73 (71.3%)
Female	29 (28.7%)
*Age (years)*	
Mean (SD)	38.4 (± 11.2)
Median (min–max)	40 (18–66)
*Education level*	
Illiterate	–
Elementary school	3 (2.9%)
Middle school	34 (33.7%)
High school	39 (38.7%)
College or more	25 (24.7%)
*Injury time (months)*	
Mean (SD)	30.4 (± 12.8)
Median (min–max)	30 (9–73)
*Level of injury*	
Cervical	38 (37.6%)
Thoracic	33 (32.7%)
Lumbar/sacral	30 (29.7%)
*ASIA grade*	
A	19 (18.8%)
B	51 (50.5%)
C	31 (30.7%)
D	–
*Bladder management*	
Spontaneous voiding	29 (28.7%)
Indwelling catheter or urostomy bag	6 (5.9%)
Condom catheter	7 (6.9%)
Intermittent catheter	59 (58.5%)

*SD* Standard deviation, *min–max* Minimum–Maximum

Of the 101 participants who completed the initial NBSS-SF questionnaire, 6 (5.9%) did not return for the retest. 4 participants (3.7%) who filled out the retest NBSS-SF questionnaire were excluded because they indicated a substantial change in bladder function between the first and second assessments, where (1) participants underwent urologic surgery, and (3) had a urinary tract infection. The test–retest reliability analysis was conducted using data from 91 participants who completed the test and retest phase. The mean overall NBSS-SF score was 11.22 ± 3.03, and the mean overall Qualiveen score was 2.04 ± 0.75.

### Reliability

The internal consistency of the NBSS-SF subdomains and the total scores were tested by Cronbach’s alpha (Table 2). When the subdomains of the NBSS-SF were tested separately, the results were 0.84 for the incontinence domain, 0.72 for the storage and voiding domain, and 0.57 for the consequences domain. However, when all of the items were assessed including the QOL question, the overall Cronbach’s alpha value for the Arabic version of the NBSS-SF was 0.82. Ninety-one patients with SCI participated in the test–retest reliability study which showed an ICC of 0.91 (95% CI 0.90–0.94, *p* < 0.001) for the overall score, and for every subdomain separately (ICC of 0.94 for incontinence, 0.72 for storage and voiding, and 0.90 for consequences) (Table 3).

**Table 2:** Internal consistency of Arabic NBSS-SF

NBSS-SF	Internal consistency (Cronbach’s α) n = 101	Cronbach’s α coefficient by Welk and al n = 230
Overall score	0.82	0.76
*Subdomains*		
Incontinence	0.84	0.86
Storage and voiding	0.72	0.71
Consequences	0.57	0.43

*NBSS-SF* Neurogenic bladder symptoms score-short from

**Table 3:** Reproducibility of Arabic NBSS-SF

NBSS-SF	Test–retest (intraclass correlation coefficient) n = 91	Intraclass correlation coefficient by Welk and al n = 120
Overall score	0.91	0.84
*Subdomains*		
Incontinence	0.94	0.80
Storage and voiding	0.72	0.80
Consequences	0.90	0.86

*NBSS-SF* Neurogenic bladder symptoms score-short from

### Validity

#### Content validity

Content validity was evaluated by two doctors (one expert in the neurologic bladder, and the other a urogynecologist) who were members of the expert committee responsible for translating the NBSS-SF into Arabic, who confirmed that the NBSS-SF covers the most relevant aspects related to NB and is appropriate for use in this subgroup of patients.

#### Construct validity

The construct validity was evaluated by performing Spearman’s rank test. There was a significant strong positive correlation between question 2 of NBSS-SF and the Qualiveen (r = 0.73, *p* < 0.001). In addition, there were also significant moderate negative correlations between question 2 of NBSS-SF with both the SF-12 mental health and physical health subdomains (r = − 0.52, *p* = 0.004 and r = − 0, 41, *p* = 0.002, respectively).

## Discussion

To employ evaluation tools designed in a foreign language, a systematic and structured procedure of validation and cultural adaptation is required to ensure the version's success while accounting for cultural and linguistic differences [23]. This procedure enables the accomplishment of standards and comparisons with worldwide data, as well as opening opportunities for multicenter trials involving other nations [33]. The NBSS-SF items were designed employing expert opinion, a literature review, and interviews with patients diagnosed with multiple sclerosis, spina bifida, and spinal cord injury [2]. As well as this instrument was created to meet clinical and research demands for a tool to measure NB symptoms and related consequences [12].

Syria is located within the high prevalence region of SCI patients due to the civil war, which caused a lot of physical, economic, and mental disorders [35]. Thus, developing an Arabic NBSS-SF version was considered necessary to monitor and evaluate the disease complications on urinary and bladder function. Most patients answered the questionnaire easily, but a few required clarifications on the content. We believe that is attributable to the extensive and rigorous procedure we followed while translating international guidelines [23]. The simplicity of usage makes the NBSS-SF a self-applicable instrument suited for evaluating the progression of the disease, even for remote patients without access to academic institutions.

The current study showed the psychometric features of the adapted NBSS-SF version (construct validity, content validity, internal consistency, and test–retest reliability). The Arabic version demonstrated an internal consistency for the total score (Cronbach’s alpha = 0.82), similar to the to the initial validation studies (Cronbach’s alpha = 0.76) [14], and French version of NBSS-SF (Cronbach’s alpha = 0.79) [21]. While Cronbach’s alpha of the three subdomains was incontinence (0.84), storage/voiding (0.72), and consequences (0.57). A similar trend was previously demonstrated by S. Berradja et al. for the French version (0.86, 0.71 and 0.43, respectively) [21], and by B. Welk et al. for the original version (0.91, 0.69 and 0.25, respectively) [14].

Internal consistency for the consequences domain was low, as suggested by Welk and colleagues [14]. This finding could be explained by variety of bladder management, where more than 50% of our population performed CIC. It is well known that using clean intermittent catheterization (CIC) in the neurogenic bladder lowers the risk of long-term complications and enhances the quality of life, whether used alone or combined with other urinary management strategies like anti-muscarinic or onabotulinum toxin [34]. According to a 2018 study by Myers and colleagues, SCI patients were more satisfied when performing CIC than with an indwelling catheter [35]. The substantial percentage of patients treated by CIC in our study population could explain the low internal consistency in the consequences domain and quality of life subscales.

Regarding test–retest reliability after a median of 10 days, we found an ICC of 0.91, similar to the validation study among a France population (0.90) [21] and high than the value of ICC in the original study (0.84) [14]. We evaluated construct validity by comparing question 2 of NBSS-SF with the Arabic version of the Short Form − 12 and the Arabic version of QoL questionnaire Qualiveen. In our study, the correlation with the Qualiveen was strong (r = 0.73), higher than the result found in the original English version (r = 0.67), which compared it with Qualiveen-SF HRQOL questionnaire [14]. Whereas the correlation with both the SF-12 mental health (r = − 0.55) and physical health (r = − 0.46) subdomains was moderate and negative, as reported in a previous study of the original version in SCI patients [19].

This study has several limitations. First, the absence of equality between the proportion of male and female participants causes limited generalizability by the proportion of male participants. Second, all patients were recruited in four academic centers in two Syrian provinces, whereas patients in rural and more remote areas may need a different approach. The third limitation of our study is the sample size. Although it is critical for study design, there is no agreement on the appropriate sample for cross-cultural adaptation and validation studies. Van Nispen et al. [36] proposed that the existing guidelines do not adequately define an appropriate sample size and suggest that because the issue is strongly reliant on the construct to be measured, researchers should make this decision and determine the adequate sample size for their studies. According to Terwe et al. [37] no guidelines have been established for the needed sample size of studies evaluating measurement. They assume that a sample size of 50 patients is sufficient to identify the minimal necessary adjustments needed to assess interpretability.

Although individuals with NB are common in rehabilitation clinics, enrolling a large sample group for studies is challenging. This difficulty is attributed to transportation and mobility restrictions making it hard for them to engage in this research study that requests multiple meetings in a short period of time [38]. Despite the study's small sample size, we were able to conduct validation and cross-cultural adaption of the NBSS-SF questionnaire for the Arabic population (Additional file 1: Appendix). The sample size in our study meets the minimum requirements outlined by Terwee et al. [37] and is similar to other validation studies [38, 39].

## Conclusion

The results of this study show that the Arabic version of the NBSS-SF has satisfactory psychometric parameters, including test–retest reliability, internal consistency, content, and construct validity. The Arabic version of the NBSS-SF is a valid and reliable instrument for assessing NB-related QOL in the Arabic population suffering from spinal cord injury. Using the NBSS-SF questionnaire in their native language will help these patients, and their physicians better understand urinary and bladder symptoms during everyday practice. The Arabic version of the NBSS-SF is suitable for research and clinical use.

## Supplementary Information

**Additional file 1:** Appendix.

## Data Availability

The original contributions presented in the study are included in the article/Additional file, further inquiries can be directed to the corresponding author.

## References

[1] Manack A (2011). Epidemiology and healthcare utilization of neurogenic bladder patients in a US claims database. Neurourol Urodyn.

[2] Clark R, Welk B (2016). Patient reported outcome measures in neurogenic bladder. Transl Androl Urol.

[3] Best KL, Ethans K, Craven BC, Noreau L, Hitzig SL (2017). Identifying and classifying quality of life tools for neurogenic bladder function after spinal cord injury: a systematic review. J Spinal Cord Med.

[4] Ginsberg D (2013). The epidemiology and pathophysiology of neurogenic bladder. Am J Manag Care.

[5] Welk B (2020). Quality of life in neurourology patients. Eur Urol Focus.

[6] Narang GL (2017). Patient-reported outcome measures in urology. Curr Opin Urol.

[7] Tamanini JTN (2004). Concurrent validity, internal consistency and responsiveness of the Portuguese version of the King’s Health Questionnaire (KHQ) in women after stress urinary incontinence surgery. Int Braz J Urol.

[8] Barry MJ (1992). The American urological association symptom index for benign prostatic hyperplasia. The measurement committee of the American urological association. J Urol.

[9] Costa P (2001). Quality of life in spinal cord injury patients with urinary difficulties. Development and validation of qualiveen. Eur Urol.

[10] Haab F (2008). Comprehensive evaluation of bladder and urethral dysfunction symptoms: development and psychometric validation of the urinary symptom profile (USP) questionnaire. Urology.

[11] Bonniaud V, Bryant D, Parratte B, Gallien P, Guyatt G (2006). Qualiveen: a urinary disorder-specific instrument for use in clinical trials in multiple sclerosis. Arch Phys Med Rehabil.

[12] Welk B, Morrow SA, Madarasz W, Potter P, Sequeira K (2013). The conceptualization and development of a patient-reported neurogenic bladder symptom score. Res Rep Urol.

[13] Welk B, Morrow S, Madarasz W, Baverstock R, Macnab J, Sequeira K (2014). The validity and reliability of the neurogenic bladder symptom score. J Urol.

[14] Welk B (2020). The creation and validation of a short form of the neurogenic bladder symptom score. Neurourol Urodyn.

[15] Cintra LKL (2019). Cross-cultural adaptation and validation of the neurogenic bladder symptom score questionnaire for Brazilian Portuguese. Int Braz J Urol.

[16] Philippova ES, Bazhenov IV, Volkova LI, Moskvina EY, Turova EL, Popova YV (2018). Russian version of the neurogenic bladder symptom score (NBSS). Urologiia.

[17] Guler MA, Doğan D, Yalcinkaya EY (2020). “Validity and reliability of the Turkish version of the neurogenic bladder symptom score. Disabil Rehabil.

[18] Tzelves L (2021). Validity and reliability of the Greek version of the neurogenic bladder symptom score (NBSS) questionnaire in a sample of Greek patients with multiple sclerosis. World J Urol.

[19] Welk B (2018). The neurogenic bladder symptom score (NBSS): a secondary assessment of its validity, reliability among people with a spinal cord injury. Spinal Cord.

[20] Pariser JJ, Welk B, Kennelly M, Elliott SP (2019). Reliability and validity of the neurogenic bladder symptom score in adults with cerebral palsy. Urology.

[21] Berradja S (2022). French version of the short form of the neurogenic bladder symptom score: cross-cultural adaptation and validation. Can Urol Assoc J J Assoc des Urol du Canada.

[22] Epstein J, Santo RM, Guillemin F (2015). A review of guidelines for cross-cultural adaptation of questionnaires could not bring out a consensus. J Clin Epidemiol.

[23] Beaton DE, Bombardier C, Guillemin F, Ferraz MB (2000). Guidelines for the process of cross-cultural adaptation of self-report measures. Spine (Phila Pa 19656).

[24] Haddad C, Sacre H, Obeid S, Salameh P, Hallit S (2021). Validation of the Arabic version of the ‘12-item short-form health survey’ (SF-12) in a sample of Lebanese adults. Arch Public Heal.

[25] El Hilali O, Eloumri A, Hajjaj-Hassouni N (2011). Qualiveen: validation of the Moroccan Arabic version of the quality of life questionnaire for spinal cord injury patients. Ann Phys Rehabil Med.

[26] Tulsky DS (2015). Overview of the spinal cord injury-quality of life (SCI-QOL) measurement system. J Spinal Cord Med.

[27] Mokkink LB (2010). The COSMIN study reached international consensus on taxonomy, terminology, and definitions of measurement properties for health-related patient-reported outcomes. J Clin Epidemiol.

[28] Keszei AP, Novak M, Streiner DL (2010). Introduction to health measurement scales. J Psychosom Res.

[29] Koo TK, Li MY (2016). A guideline of selecting and reporting intraclass correlation coefficients for reliability research. J Chiropr Med.

[30] Sousa VD, Rojjanasrirat W (2011). Translation, adaptation and validation of instruments or scales for use in cross-cultural health care research: a clear and user-friendly guideline. J Eval Clin Pract.

[31] George D. SPSS for Windows step by step: a simple guide and reference. (2003).

[32] Schober P, Boer C, Schwarte LA (2018). Correlation coefficients: appropriate use and interpretation. Anesth Analg.

[33] Guillemin F, Bombardier C, Beaton D (1993). Cross-cultural adaptation of health-related quality of life measures: literature review and proposed guidelines. J Clin Epidemiol.

[34] Di Benedetto P (2011). Clean intermittent self-catheterization in neuro-urology. Eur J Phys Rehabil Med.

[35] Myers JB (2019). Patient reported bladder related symptoms and quality of life after spinal cord injury with different bladder management strategies. J Urol.

[36] van Nispen RMA, Knol DL, Mokkink LB, Comijs HC, Deeg DJH, van Rens GHMB (2010). Vision-related quality of life core measure (VCM1) showed low-impact differential item functioning between groups with different administration modes. J Clin Epidemiol.

[37] Terwee CB (2007). Quality criteria were proposed for measurement properties of health status questionnaires. J Clin Epidemiol.

[38] D’Ancona CAL (2009). Quality of life of neurogenic patients: translation and validation of the Portuguese version of Qualiveen. Int Urol Nephrol.

[39] Calado AA (2010). Cross-cultural adaptation of the dysfunctional voiding score symptom (DVSS) questionnaire for Brazilian children. Int Braz J Urol.

