# Supplementary material for: The validity and reliability of the Arabic version of the short form of neurogenic bladder symptoms score in patients with spinal cord injury
Source: J Orthop Surg Res. 2023 Jun 27;18:464. doi: 10.1186/s13018-023-03956-6 (PMC10303291; doi:10.1186/s13018-023-03956-6)
Supplement: Supplementary file 1 — Additional file 1: Appendix. [file 13018_2023_3956_MOESM1_ESM.docx]

**The Neurogenic Bladder Symptom Score Short Form**

- These questions ask about urinary problems that you may have.
- Please answer all questions, and choose only one answer for each question.
- It should take 1‐2 minutes to complete this questionnaire.
- Please think about your usual bladder function when you answer the questions. Do not include temporary changes to your bladder function, such as a recent urinary tract infection.
- All of the questions can be answered by patients who urinate on their own, use catheters, have had bladder surgery, or use urine collection devices.

1. Most of the time I manage my bladder or urination:
2. With a catheter in all the time, or a urostomy bag
3. With a condom catheter
4. With an intermittent catheter
5. By urinating mostly into the toilet
6. By urinating mostly into diapers/pads
7. If you had to live the rest of your life with the way your bladder (or urinary reservoir) currently works, how would you feel?
8. Pleased
9. Mostly satisfied
10. Mixed: equally satisfied and unsatisfied
11. Mostly Unsatisfied
12. Unhappy
13. During the day, how often do you have urine leakage (including leakage around a catheter or stoma):
14. Zero‐don’t have urine leakage
15. Rarely
16. A few times a week
17. About once a day
18. More than once a day
19. During the day, the amount of urine leakage (including leakage around a catheter or stoma):
20. Is zero‐I don’t have urine leakage
21. Is minimal and doesn’t require pads
22. Requires 1 pad
23. Requires 2 pads
24. Requires 3 or more pads
25. During the day, the longest time I can usually stay dry without any urine leakage is
26. This isn’t an issue for me. I don’t have urine leakage
27. More than 3 hours
28. About 2‐3 hours
29. About 1‐2 hours
30. Less than an hour
31. The sudden urge to urinate, (or a bladder spasm) occurs:
32. Never
33. Rarely
34. A few times a day
35. Many times, a day
36. When I need to urinate (or use an intermittent catheter):
37. I don’t think about urinating. I have a catheter or stoma bag
38. I can do this when it is convenient without leaking urine
39. I can only delay this a few minutes or I may leak urine
40. I have to do this right away or I may leak urine
41. During the day, the longest I can usually go between urinating, using a catheter, or emptying my urine bag is:
42. More than 3 hours
43. More than 2 hours but less than 3 hours
44. About 1‐2 hours
45. Less than an hour
46. I have a urinary tract infection with symptoms (for example pain, foul-smelling urine, fever):
47. Never
48. About once a year or less
49. A few times a year
50. Once every few months
51. Once a month, or more
52. I find the pills or medications I use for my urination or bladder are:
53. I don’t take pills or medications for my bladder
54. Effective
55. Partially effective
56. Not very effective
